# Updated Genome Sequence for the Probiotic Bacterium Bifidobacterium animalis subsp. *lactis* BB-12

**DOI:** 10.1128/MRA.00078-21

**Published:** 2021-07-08

**Authors:** Kristian Jensen, Kosai Al-Nakeeb, Anna Koza, Ahmad A. Zeidan

**Affiliations:** a Systems Biology, Discovery, Chr. Hansen A/S, Hørsholm, Denmark; University of Maryland School of Medicine

## Abstract

The genome of Bifidobacterium animalis subsp. *lactis* BB-12 was sequenced using Oxford Nanopore Technologies long-read and Illumina short-read sequencing platforms. A hybrid genome assembly approach was used to construct an updated complete genome sequence for BB-12 containing 1,944,152 bp, with a G+C content of 60.5% and 1,615 genes.

## ANNOUNCEMENT

Bifidobacteria are often used as probiotics due to their health-promoting effects (1). Bifidobacterium animalis subsp. *lactis* BB-12 (2) has been associated with positive effects in areas such as immune function (3–5), gastrointestinal health (6), and the respiratory system (7, 8). Here, we present a second version of the BB-12 genome, generated through state-of-the-art sequencing technologies using both Illumina and Oxford Nanopore Technologies (ONT) sequencing platforms.

BB-12 (DSM 15954) was obtained from the German Collection of Microorganisms and Cell Cultures (DSMZ) (Braunschweig, Germany) in April 2004 and stored at −80°C. Cells were grown anaerobically at 37°C in MRS broth containing 0.05% l-cysteine hydrochloride monohydrate. Genomic DNA was extracted with the DNeasy blood and tissue kit on a QiaCube system (Qiagen, Germany). Sequencing libraries were generated using a KAPA HyperPlus library preparation kit and KAPA dual-indexed adapters (Roche, Switzerland), and AMPure XP beads (Beckman Coulter, USA) were used for library cleanup steps, following the manufacturers’ protocols. DNA concentrations were measured on a Qubit 3.0 fluorimeter using Qubit double-stranded DNA (dsDNA) broad-range and Qubit 1X dsDNA high-sensitivity (HS) assays (Thermo Fisher Scientific, USA). The average dsDNA library size distribution was determined using the Agilent HS next-generation sequencing (NGS) fragment (1 to 6,000 bp) kit on an Agilent fragment analyzer. Libraries were sequenced with the 600-cycle MiSeq reagent kit v3 on a MiSeq platform (Illumina Inc., USA) with a paired-end protocol and read lengths of 301 nucleotides (nt). Short-read sequencing yielded 686,356 read pairs with a total length of 255,784,736 nt. The reads were trimmed for quality using AdapterRemoval v2.2.4 (9) with parameters --minquality 20 --minlength 30 --trimqualities --trimns --trim5p 15.

For long-read sequencing, genomic DNA was extracted with a Genomic Maxi AX gravity column-based kit (A&A Biotechnology, Poland), prepared using a rapid barcoding sequencing kit (SQK-RBK004; ONT), and sequenced on a MinION flow cell (R9.4.1 FLO-MIN106; ONT) for 48 h. This yielded 72,175 reads with a total length of 374,342,567 nt and an *N*_50_ value of 10,185 nt. Base calling was performed with the Guppy base caller v3.2.10, and adapters were trimmed using ONT MinKNOW software.

The complete genome sequence of BB-12 was assembled using the long and short sequencing reads with Unicycler v0.4.7 (10) in conservative mode, resulting in one 1,944,152-bp circular chromosome with a G+C content of 60.5%. The genome sequence was annotated with Prokaryotic Genome Annotation Pipeline (PGAP) v4.13 (11), resulting in 1,615 genes.

To assess whether the genome sequenced here differs from the previously available BB-12 genome (GenBank accession number NC_017214.1), breseq v0.35.5 (12) with default settings was used to map Illumina reads from this work to both genome sequences. Mapping of Illumina reads to the genome sequence produced in this work did not yield any variants, while mapping to the previously available genome sequence resulted in 74 single-nucleotide differences and 235 small indels (Table 1).

**TABLE 1:** Overview of the genetic differences identified by comparing the newly generated reads to the previously available BB12 genome sequence (GenBank accession number NC_017214.1)^*a*^

Nucleotide position	Mutation	Annotation	Gene(s)
469	C→A	Q49K (CAA→AAG)	*BIF_00469* →
471	A→G	Q49K (CAA→AAG)	*BIF_00469* →
21536	+A	Coding (867/927 nt)	*BIF_02142* →
21540	3 bp→12 bp	Coding (871–873/927 nt)	*BIF_02142* →
28398	+C	Intergenic (+28/−48)	*BIF_00002* → / → *BIF_02140*
28414	A→T	Intergenic (+44/−32)	*BIF_00002* → / → *BIF_02140*
28417	4 bp→18 bp	Intergenic (+47/−26)	*BIF_00002* → / → *BIF_02140*
45995	+TGGAATTCCACGGGCCT	Intergenic (+95/+86)	*BIF_01763* → / ← *BIF_01079*
93265	+CG	Intergenic (+43/+6)	*BIF_01365* → / ← *BIF_02171*
93267	+AACC	Intergenic (+45/+4)	*BIF_01365* → / ← *BIF_02171*
93268	T→A	Intergenic (+46/+3)	*BIF_01365* → / ← *BIF_02171*
93270	+AT	Intergenic (+48/+1)	*BIF_01365* → / ← *BIF_02171*
103949	4 bp→22 bp	Intergenic (+18/+56)	*BIF_01186* → / ← *BIF_02012*
103961	C→A	Intergenic (+30/+47)	*BIF_01186* → / ← *BIF_02012*
103963	Δ1 bp	Intergenic (+32/+45)	*BIF_01186* → / ← *BIF_02012*
112686	+25 bp	Intergenic (−226/+31)	*BIF_00107* ← / ← *BIF_00801*
132978	5 bp→19 bp	Intergenic (+14/−185)	*BIF_01865* → / → *BIF_02174*
132990	+G	Intergenic (+26/−177)	*BIF_01865* → / → *BIF_02174*
132999	+C	Intergenic (+35/−168)	*BIF_01865* → / → *BIF_02174*
152141	21 bp→37 bp	Intergenic (+39/+16)	*BIF_01884* → / ← *BIF_00858*
156687	+23 bp	Intergenic (+51/+2)	*BIF_00548* → / ← *BIF_00217*
174620	+G	Coding (9/300 nt)	*BIF_02178* →
174628	+G	Coding (17/300 nt)	*BIF_02178* →
174796	3 bp→25 bp	Coding (185–187/300 nt)	*BIF_02178* →
174847	+G	Coding (236/300 nt)	*BIF_02178* →
174849	G→C	A80P (GCC→CCC)	*BIF_02178* →
226865	+A	Coding (974/1,167 nt)	*BIF_00004* ←
226867	G→C	S324S (TCC→TCG)	*BIF_00004* ←
303227	+GGTGG	Intergenic (+30/+242)	*BIF_01290* → / ← *BIF_00983*
332638	+GCATGCGGCGA	Intergenic (+65/+62)	*BIF_00009* → / ← *BIF_01572*
332642	G→C	Intergenic (+69/+58)	*BIF_00009* → / ← *BIF_01572*
348109	9 bp→32 bp	Intergenic (+13/+31)	*BIF_01159* → / ← *BIF_02184*
374215	+32 bp	Intergenic (−158/+189)	*BIF_00055* ← / ← *BIF_01897*
374264	+G	Intergenic (−207/+140)	*BIF_00055* ← / ← *BIF_01897*
374286	2 bp→GC	Intergenic (−229/+117)	*BIF_00055* ← / ← *BIF_01897*
395152	49 bp→66 bp	Intergenic (+77/+63)	*BIF_01176* → / ← *BIF_00941*
395195	7 bp→19 bp	Intergenic (+120/+62)	*BIF_01176* → / ← *BIF_00941*
412279	+CTGAGCACACGGGGGCCG	Intergenic (+8/−75)	*BIF_01519* → / → *BIF_01115*
412281	G→T	Intergenic (+10/−73)	*BIF_01519* → / → *BIF_01115*
412292	+A	Intergenic (+21/−62)	*BIF_01519* → / → *BIF_01115*
412906	+54 bp	Intergenic (+25/+20)	*BIF_01115* → / ← *BIF_01114*
416028	+C	Intergenic (+371/+25)	*BIF_01112* → / ← *BIF_02188*
416031	+GA	Intergenic (+374/+22)	*BIF_01112* → / ← *BIF_02188*
423866	1 bp→GG	Intergenic (+45/+26)	*BIF_00671* → / ← *BIF_00700*
423877	1 bp→18 bp	Intergenic (+56/+15)	*BIF_00671* → / ← *BIF_00700*
426071	+GGCGCCACACGCGAA	Intergenic (−149/+31)	*BIF_00700* ← / ← *BIF_01226*
426073	+C	Intergenic (−151/+29)	*BIF_00700* ← / ← *BIF_01226*
442719	Δ1 bp	Intergenic (−50/+31)	*BIF_01320* ← / ← *BIF_00999*
442725	6 bp→19 bp	Intergenic (−56/+20)	*BIF_01320* ← / ← *BIF_00999*
458009	+45 bp	Coding (13/132 nt)	*BIF_02190* ←
479759	A→G	Intergenic (+44/+44)	*BIF_01252* → / ← *BIF_01632*
512389	2 bp→GC	Coding (58–59/180 nt)	*BIF_02194* ←
512406	+C	Coding (42/180 nt)	*BIF_02194* ←
512414	+C	Coding (34/180 nt)	*BIF_02194* ←
515142	2 bp→AA	Coding (116–117/2,316 nt)	*BIF_02061* →
515145	G→A	W40* (TGG→TAG)	*BIF_02061* →
515153	G→C	V43L (GTA→CTA)	*BIF_02061* →
544458	A→G	T524T (ACA→ACG)	*BIF_01621* →
544491	20 bp→35 bp	Coding (1,605–1,624/1,653 nt)	*BIF_01621* →
551925	3 bp→17 bp	Coding (31–33/1,518 nt)	*BIF_02058* →
551931	Δ1 bp	Coding (37/1,518 nt)	*BIF_02058* →
555527	62 bp→77 bp	Intergenic (+44/+177)	*BIF_00496* → / ← *BIF_01682*
574023	+AACCCGCCC	Intergenic (−880/+30)	*BIF_16SrRNA12* ← / ← *BIF_01549*
601285	Δ1 bp	Coding (2/771 nt)	*BIF_00943* ←
607329	+AC	Coding (148/159 nt)	*BIF_02197* →
607331	G→C	A50P (GCG→CCC)	*BIF_02197* →
627558	Δ1 bp	Intergenic (+54/−170)	*BIF_01669* → / → *BIF_02198*
627563	+CCCGCCGGC	Intergenic (+59/−165)	*BIF_01669* → / → *BIF_02198*
631554	+C	Intergenic (−383/+213)	*BIF_00740* ← / ← *BIF_00658*
631589	+A	Intergenic (−418/+178)	*BIF_00740* ← / ← *BIF_00658*
631599	Δ1 bp	Intergenic (−428/+168)	*BIF_00740* ← / ← *BIF_00658*
631605	11 bp→48 bp	Intergenic (−434/+152)	*BIF_00740* ← / ← *BIF_00658*
632722	+24 bp	Intergenic (−212/+219)	*BIF_00658* ← / ← *BIF_01209*
632782	+G	Intergenic (−272/+159)	*BIF_00658* ← / ← *BIF_01209*
690916	7 bp→28 bp	Intergenic (+28/+71)	*BIF_01558* → / ← *BIF_01474*
795160	(G)_8→9_	Coding (834/1,299 nt)	*BIF_01420* →
803347	+AGGTGGGG	Coding (433/435 nt)	*BIF_02002* ←
803348	+AT	Coding (432/435 nt)	*BIF_02002* ←
803350	A→C	S144A (TCG→GCA)	*BIF_02002* ←
805769	+AGCA	Intergenic (−41/+271)	*BIF_00477* ← / ← *BIF_02124*
813756	+C	Intergenic (+67/−63)	*BIF_01190* → / → *BIF_02126*
889981	48 bp→76 bp	Intergenic (+42/+30)	*BIF_01494* → / ← *BIF_00763*
890037	+G	Intergenic (+98/+21)	*BIF_01494* → / ← *BIF_00763*
899064	+A	Intergenic (+234/−448)	*BIF_01023* → / → *BIF_01805*
899066	C→A	Intergenic (+236/−446)	*BIF_01023* → / → *BIF_01805*
899080	+G	Intergenic (+250/−432)	*BIF_01023* → / → *BIF_01805*
899096	C→A	Intergenic (+266/−416)	*BIF_01023* → / → *BIF_01805*
927296	+GGG	Intergenic (+878/+33)	*BIF_01509* → / ← *BIF_01083*
980962	+G	Intergenic (−141/+180)	*BIF_00863* ← / ← *BIF_01247*
996014	1 bp→CG	Intergenic (+36/−287)	*BIF_02104* → / → *BIF_01142*
1054723	+29 bp	Intergenic (+134/−290)	*BIF_00088* → / → *BIF_02096*
1054848	2 bp→21 bp	Intergenic (+259/−164)	*BIF_00088* → / → *BIF_02096*
1054883	2 bp→GT	Intergenic (+294/−129)	*BIF_00088* → / → *BIF_02096*
1151595	+C	Intergenic (+39/+27)	*BIF_02003* → / ← *BIF_tRNA28*
1203020	(C)_6→5_	Intergenic (−170/−29)	*BIF_02234* ← / → *BIF_00647*
1203058	(G)_6→5_	Coding (10/2,550 nt)	*BIF_00647* →
1249797	15 bp→34 bp	Coding (32–46/126 nt)	*BIF_02237* →
1254633	T→C	K65R (AAG→AGG)	*BIF_00633* ←
1270898	+T	Intergenic (+15/+58)	*BIF_00472* → / ← *BIF_00205*
1270906	+TGTGGGGCCCTACGG	Intergenic (+23/+50)	*BIF_00472* → / ← *BIF_00205*
1270910	+C	Intergenic (+27/+46)	*BIF_00472* → / ← *BIF_00205*
1283590	+TTCGGG	Intergenic (+24/−367)	*BIF_00825* → / → *BIF_00906*
1283592	+CC	Intergenic (+26/−365)	*BIF_00825* → / → *BIF_00906*
1291704	2 bp→11 bp	Intergenic (−1,307/+149)	*BIF_01039* ← / ← *BIF_00316*
1291708	+T	Intergenic (−1,311/+146)	*BIF_01039* ← / ← *BIF_00316*
1291709	G→C	Intergenic (−1,312/+145)	*BIF_01039* ← / ← *BIF_00316*
1309076	+C	Coding (128/1,077 nt)	*BIF_01009* ←
1309114	A→G	C30C (TGT→TGC)	*BIF_01009* ←
1333145	+CC	Intergenic (+76/+16)	*BIF_01182* → / ← *BIF_00625*
1333149	+ACGCA	Intergenic (+80/+12)	*BIF_01182* → / ← *BIF_00625*
1333151	+A	Intergenic (+82/+10)	*BIF_01182* → / ← *BIF_00625*
1333153	+C	Intergenic (+84/+8)	*BIF_01182* → / ← *BIF_00625*
1345841	+A	Intergenic (+107/+54)	*BIF_01862* → / ← *BIF_00279*
1345851	3 bp→18 bp	Intergenic (+117/+42)	*BIF_01862* → / ← *BIF_00279*
1345877	+G	Intergenic (+143/+18)	*BIF_01862* → / ← *BIF_00279*
1345918	+G	Coding (517/540 nt)	*BIF_00279* ←
1345930	+T	Coding (505/540 nt)	*BIF_00279* ←
1347684	+ACC	Intergenic (−32/−35)	*BIF_02123* ← / → *BIF_00396*
1347686	+C	Intergenic (−34/−33)	*BIF_02123* ← / → *BIF_00396*
1347687	+C	Intergenic (−35/−32)	*BIF_02123* ← / → *BIF_00396*
1352581	G→A	Intergenic (+158/+131)	*BIF_00072* → / ← *BIF_00341*
1352652	+ACAGAAGGGCGGT	Intergenic (+229/+60)	*BIF_00072* → / ← *BIF_00341*
1352657	C→G	Intergenic (+234/+55)	*BIF_00072* → / ← *BIF_00341*
1352659	A→C	Intergenic (+236/+53)	*BIF_00072* → / ← *BIF_00341*
1360042	19 bp→35 bp	Intergenic (+28/+7)	*BIF_00368* → / ← *BIF_01760*
1376069	14 bp→33 bp	Intergenic (+38/+27)	*BIF_01846* → / ← *BIF_00467*
1414041	C→A	A373D (GCC→GAC)	*BIF_00934* →
1414072	Δ1 bp	Coding (1,149/1,152 nt)	*BIF_00934* →
1414079	+G	Intergenic (+4/+74)	*BIF_00934* → / ← *BIF_00718*
1414090	8 bp→28 bp	Intergenic (+15/+56)	*BIF_00934* → / ← *BIF_00718*
1419213	+G	Intergenic (+37/+331)	*BIF_00332* → / ← *BIF_02065*
1419441	T→G	Intergenic (+265/+103)	*BIF_00332* → / ← *BIF_02065*
1419443	C→T	Intergenic (+267/+101)	*BIF_00332* → / ← *BIF_02065*
1419447	40 bp→53 bp	Intergenic (+271/+58)	*BIF_00332* → / ← *BIF_02065*
1419488	T→G	Intergenic (+312/+56)	*BIF_00332* → / ← *BIF_02065*
1419490	+TG	Intergenic (+314/+54)	*BIF_00332* → / ← *BIF_02065*
1419492	1 bp→AC	Intergenic (+316/+52)	*BIF_00332* → / ← *BIF_02065*
1419499	2 bp→GC	Intergenic (+323/+44)	*BIF_00332* → / ← *BIF_02065*
1419504	+C	Intergenic (+328/+40)	*BIF_00332* → / ← *BIF_02065*
1424409	4 bp→36 bp	Intergenic (+52/−47)	*BIF_00028* → / → *BIF_00780*
1429003	1 bp→16 bp	Intergenic (+54/−44)	*BIF_00776* → / → *BIF_01792*
1429006	T→G	Intergenic (+57/−41)	*BIF_00776* → / → *BIF_01792*
1429008	C→T	Intergenic (+59/−39)	*BIF_00776* → / → *BIF_01792*
1435740	10 bp→24 bp	Intergenic (+33/+22)	*BIF_00308* → / ← *BIF_00385*
1435753	C→A	Intergenic (+46/+18)	*BIF_00308* → / ← *BIF_00385*
1442201	+G	Intergenic (+56/+22)	*BIF_00264* → / ← *BIF_01752*
1444091	1 bp→35 bp	Intergenic (−135/+100)	*BIF_01751* ← / ← *BIF_01116*
1444149	+C	Intergenic (−193/+42)	*BIF_01751* ← / ← *BIF_01116*
1444153	+C	Intergenic (−197/+38)	*BIF_01751* ← / ← *BIF_01116*
1459986	14 bp→30 bp	Intergenic (+38/+3)	*BIF_00179* → / ← *BIF_00879*
1466361	+TTGCGTTCCC	Intergenic (−140/+28)	*BIF_01803* ← / ← *BIF_00130*
1466364	+C	Intergenic (−143/+25)	*BIF_01803* ← / ← *BIF_00130*
1466365	G→T	Intergenic (−144/+24)	*BIF_01803* ← / ← *BIF_00130*
1469891	+G	Intergenic (+56/−504)	*BIF_00011* → / → *BIF_02078*
1469893	+G	Intergenic (+58/−502)	*BIF_00011* → / → *BIF_02078*
1470155	A→C	Intergenic (+320/−240)	*BIF_00011* → / → *BIF_02078*
1470159	T→C	Intergenic (+324/−236)	*BIF_00011* → / → *BIF_02078*
1470162	+C	Intergenic (+327/−233)	*BIF_00011* → / → *BIF_02078*
1470165	T→C	Intergenic (+330/−230)	*BIF_00011* → / → *BIF_02078*
1470171	G→C	Intergenic (+336/−224)	*BIF_00011* → / → *BIF_02078*
1470174	G→C	Intergenic (+339/−221)	*BIF_00011* → / → *BIF_02078*
1470177	+C	Intergenic (+342/−218)	*BIF_00011* → / → *BIF_02078*
1470183	1 bp→CC	Intergenic (+348/−212)	*BIF_00011* → / → *BIF_02078*
1470187	+C	Intergenic (+352/−208)	*BIF_00011* → / → *BIF_02078*
1470190	T→C	Intergenic (+355/−205)	*BIF_00011* → / → *BIF_02078*
1470192	T→C	Intergenic (+357/−203)	*BIF_00011* → / → *BIF_02078*
1470195	T→C	Intergenic (+360/−200)	*BIF_00011* → / → *BIF_02078*
1470204	T→C	Intergenic (+369/−191)	*BIF_00011* → / → *BIF_02078*
1470206	A→T	Intergenic (+371/−189)	*BIF_00011* → / → *BIF_02078*
1470216	G→C	Intergenic (+381/−179)	*BIF_00011* → / → *BIF_02078*
1470227	2 bp→GC	Intergenic (+392/−167)	*BIF_00011* → / → *BIF_02078*
1470235	G→T	Intergenic (+400/−160)	*BIF_00011* → / → *BIF_02078*
1470237	T→C	Intergenic (+402/−158)	*BIF_00011* → / → *BIF_02078*
1470245	T→C	Intergenic (+410/−150)	*BIF_00011* → / → *BIF_02078*
1470253	A→C	Intergenic (+418/−142)	*BIF_00011* → / → *BIF_02078*
1470256	T→C	Intergenic (+421/−139)	*BIF_00011* → / → *BIF_02078*
1470259	T→C	Intergenic (+424/−136)	*BIF_00011* → / → *BIF_02078*
1470276	T→C	Intergenic (+441/−119)	*BIF_00011* → / → *BIF_02078*
1470279	4 bp→CATT	Intergenic (+444/−113)	*BIF_00011* → / → *BIF_02078*
1470308	4 bp→CCG	Intergenic (+473/−84)	*BIF_00011* → / → *BIF_02078*
1470314	T→C	Intergenic (+479/−81)	*BIF_00011* → / → *BIF_02078*
1470322	A→C	Intergenic (+487/−73)	*BIF_00011* → / → *BIF_02078*
1470328	T→C	Intergenic (+493/−67)	*BIF_00011* → / → *BIF_02078*
1474821	+G	Coding (7/2,349 nt)	*BIF_00363* →
1474832	+C	Coding (18/2,349 nt)	*BIF_00363* →
1494471	+TGAAGCGGC	Intergenic (+15/+59)	*BIF_00354* → / ← *BIF_02081*
1494477	T→G	Intergenic (+21/+53)	*BIF_00354* → / ← *BIF_02081*
1494479	1 bp→TG	Intergenic (+23/+51)	*BIF_00354* → / ← *BIF_02081*
1507139	7 bp→21 bp	Intergenic (+39/+35)	*BIF_00355* → / ← *BIF_00882*
1516347	Δ1 bp	Intergenic (+37/−88)	*BIF_01797* → / → *BIF_tRNA37*
1516350	+TC	Intergenic (+40/−85)	*BIF_01797* → / → *BIF_tRNA37*
1533794	C→A	Intergenic (−79/+25)	*BIF_00836* ← / ← *BIF_00445*
1533800	3 bp→15 bp	Intergenic (−85/+17)	*BIF_00836* ← / ← *BIF_00445*
1537535	G→A	Intergenic (+448/−131)	*BIF_01766* → / → *BIF_02245*
1537566	C→G	Intergenic (+479/−100)	*BIF_01766* → / → *BIF_02245*
1537568	G→C	Intergenic (+481/−98)	*BIF_01766* → / → *BIF_02245*
1537618	C→T	Intergenic (+531/−48)	*BIF_01766* → / → *BIF_02245*
1537650	2 bp→AT	Intergenic (+563/−15)	*BIF_01766* → / → *BIF_02245*
1537653	1 bp→CG	Intergenic (+566/−13)	*BIF_01766* → / → *BIF_02245*
1537688	A→C	N8T (AAC→ACC)	*BIF_02245* →
1537694	A→T	E10V (GAG→GTG)	*BIF_02245* →
1537708	C→T	P15S (CCT→TCC)	*BIF_02245* →
1537710	T→C	P15S (CCT→TCC)	*BIF_02245* →
1537719	2 bp→GC	Coding (54–55/99 nt)	*BIF_02245* →
1537727	Δ1 bp	Coding (62/99 nt)	*BIF_02245* →
1537733	+TG	Coding (68/99 nt)	*BIF_02245* →
1537743	G→C	M26I (ATG→ATC)	*BIF_02245* →
1541799	Δ1 bp	Coding (970/1,029 nt)	*BIF_02111* →
1541804	69 bp→84 bp		*[BIF_02111]*
1541874	+C	Intergenic (+16/−107)	*BIF_02111* → / → *BIF_01915*
1576870	+C	Intergenic (+277/+73)	*BIF_00659* → / ← *BIF_00506*
1576890	2 bp→10 bp	Intergenic (+297/+52)	*BIF_00659* → / ← *BIF_00506*
1589406	+ATGCGCCTGAC	Intergenic (−86/+44)	*BIF_01830* ← / ← *BIF_00522*
1593487	2 bp→GC	Intergenic (−92/+36)	*BIF_02016* ← / ← *BIF_02017*
1593511	+C	Intergenic (−116/+13)	*BIF_02016* ← / ← *BIF_02017*
1593523	+G	Intergenic (−128/+1)	*BIF_02016* ← / ← *BIF_02017*
1593561	+T	Coding (242/279 nt)	*BIF_02017* ←
1595922	12 bp→22 bp	Intergenic (+63/+15)	*BIF_00337* → / ← *BIF_01744*
1595936	+GA	Intergenic (+77/+12)	*BIF_00337* → / ← *BIF_01744*
1595981	Δ1 bp	Coding (846/879 nt)	*BIF_01744* ←
1595984	+AC	Coding (843/879 nt)	*BIF_01744* ←
1595988	+C	Coding (839/879 nt)	*BIF_01744* ←
1602531	3 bp→13 bp	Intergenic (+9/−74)	*BIF_00963* → / → *BIF_00287*
1602535	1 bp→CC	Intergenic (+13/−72)	*BIF_00963* → / → *BIF_00287*
1610137	Δ2 bp	Intergenic (−9/+26)	*BIF_01901* ← / ← *BIF_00277*
1634778	+C	Intergenic (+137/−53)	*BIF_01000* → / → *BIF_00897*
1657183	12 bp→30 bp	Intergenic (−58/+30)	*BIF_01871* ← / ← *BIF_00406*
1671395	8 bp→29 bp	Intergenic (+22/+60)	*BIF_00122* → / ← *BIF_00441*
1676721	+CGGGAGCCTTCCCATATCAA	Intergenic (+49/+5)	*BIF_00162* → / ← *BIF_00129*
1685544	12 bp→38 bp	Intergenic (+9/+81)	*BIF_00244* → / ← *BIF_01784*
1685646	Δ1 bp	Coding (515/525 nt)	*BIF_01784* ←
1691786	2 bp→16 bp	Intergenic (−152/+40)	*BIF_00694* ← / ← *BIF_00758*
1704247	+G	Intergenic (+37/+72)	*BIF_00639* → / ← *BIF_01823*
1704249	38 bp→61 bp	Intergenic (+39/+33)	*BIF_00639* → / ← *BIF_01823*
1704394	+G	Coding (414/489 nt)	*BIF_01823* ←
1714609	+ATACGAAGAGGCCC	Intergenic (+15/+75)	*BIF_02095* → / ← *BIF_02255*
1714612	A→G	Intergenic (+18/+72)	*BIF_02095* → / ← *BIF_02255*
1714619	2 bp→TG	Intergenic (+25/+64)	*BIF_02095* → / ← *BIF_02255*
1714622	G→C	Intergenic (+28/+62)	*BIF_02095* → / ← *BIF_02255*
1720652	14 bp→29 bp		*[BIF_02256]*
1735437	17 bp→34 bp	Intergenic (−31/+299)	*BIF_01975* ← / ← *BIF_00492*
1747481	Δ1 bp	Intergenic (+38/+25)	*BIF_00693* → / ← *BIF_00329*
1747488	+A	Intergenic (+45/+18)	*BIF_00693* → / ← *BIF_00329*
1747489	+CCCCTCACATTT	Intergenic (+46/+17)	*BIF_00693* → / ← *BIF_00329*
1755127	C→G	M12I (ATG→ATC)	*BIF_00612* ←
1755177	+C	Coding (2,001/2,019 nt)	*BIF_00102* ←
1755247	2 bp→GC	Coding (1,930–1,931/2,019 nt)	*BIF_00102* ←
1756978	+G	Coding (200/2,019 nt)	*BIF_00102* ←
1757068	1 bp→GT	Coding (110/2,019 nt)	*BIF_00102* ←
1757069	+C	Coding (109/2,019 nt)	*BIF_00102* ←
1759022	+A	Intergenic (−66/+70)	*BIF_tRNA49* ← / ← *BIF_00125*
1759023	+ATGGGGTGT	Intergenic (−67/+69)	*BIF_tRNA49* ← / ← *BIF_00125*
1759024	+C	Intergenic (−68/+68)	*BIF_tRNA49* ← / ← *BIF_00125*
1759026	G→T	Intergenic (−70/+66)	*BIF_tRNA49* ← / ← *BIF_00125*
1759057	+C	Intergenic (−101/+35)	*BIF_tRNA49* ← / ← *BIF_00125*
1770206	7 bp→20 bp	Intergenic (+28/+64)	*BIF_01817* → / ← *BIF_01834*
1770224	G→C	Intergenic (+46/+52)	*BIF_01817* → / ← *BIF_01834*
1787741	18 bp→50 bp	Intergenic (+6/+30)	*BIF_02258* → / ← *BIF_00170*
1790308	Δ1 bp	Intergenic (+24/+44)	*BIF_01753* → / ← *BIF_01191*
1790315	+GGGCCCGCGAACAC	Intergenic (+31/+37)	*BIF_01753* → / ← *BIF_01191*
1790322	Δ1 bp	Intergenic (+38/+30)	*BIF_01753* → / ← *BIF_01191*
1790332	+G	Intergenic (+48/+20)	*BIF_01753* → / ← *BIF_01191*
1799928	6 bp→16 bp	Intergenic (+109/−49)	*BIF_02068* → / → *BIF_00066*
1800775	2 bp→TA	Intergenic (+95/+64)	*BIF_00066* → / ← *BIF_01169*
1800778	A→C	Intergenic (+98/+62)	*BIF_00066* → / ← *BIF_01169*
1800781	Δ1 bp	Intergenic (+101/+59)	*BIF_00066* → / ← *BIF_01169*
1800786	+C	Intergenic (+106/+54)	*BIF_00066* → / ← *BIF_01169*
1800822	+AG	Intergenic (+142/+18)	*BIF_00066* → / ← *BIF_01169*
1800824	6 bp→14 bp	Intergenic (+144/+11)	*BIF_00066* → / ← *BIF_01169*
1800831	+CC	Intergenic (+151/+9)	*BIF_00066* → / ← *BIF_01169*
1805672	13 bp→37 bp	Intergenic (−25/+12)	*BIF_01167* ← / ← *BIF_02259*
1806026	+G	Coding (2,837/2,883 nt)	*BIF_00729* ←
1809002	Δ1 bp	Coding (192/1,608 nt)	*BIF_00491* →
1809008	+G	Coding (198/1,608 nt)	*BIF_00491* →
1809045	25 bp→59 bp	Coding (235–259/1,608 nt)	*BIF_00491* →
1815438	+GGAAGGGGC	Intergenic (−7/+29)	*BIF_00256* ← / ← *BIF_00796*
1815443	A→C	Intergenic (−12/+24)	*BIF_00256* ← / ← *BIF_00796*
1815446	Δ1 bp	Intergenic (−15/+21)	*BIF_00256* ← / ← *BIF_00796*
1815449	Δ1 bp	Intergenic (−18/+18)	*BIF_00256* ← / ← *BIF_00796*
1824017	+26 bp	Intergenic (+34/+19)	*BIF_00792* → / ← *BIF_01147*
1832709	T→A	R51W (AGG→TGG)	*BIF_02260* ←
1832733	Δ1 bp	Coding (127/180 nt)	*BIF_02260* ←
1832760	Δ1 bp	Coding (100/180 nt)	*BIF_02260* ←
1850719	3 bp→13 bp	Intergenic (−100/+17)	*BIF_00078* ← / ← *BIF_01879*
1851378	+T	Coding (71/711 nt)	*BIF_01879* ←
		Coding (163/216 nt)	*BIF_02261* ←
1853256	Δ1 bp	Coding (63/195 nt)	*BIF_02082* ←
1885096	10 bp→24 bp	Intergenic (+17/+55)	*BIF_01746* → / ← *BIF_01789*
1885634	2 bp→GC	Coding (1,865–1,866/2,340 nt)	*BIF_01789* ←
1885679	2 bp→CT	Coding (1,820–1,821/2,340 nt)	*BIF_01789* ←
1885686	+G	Coding (1,814/2,340 nt)	*BIF_01789* ←
1885711	+G	Coding (1,789/2,340 nt)	*BIF_01789* ←
1885722	+A	Coding (1,778/2,340 nt)	*BIF_01789* ←
1885787	2 bp→GC	Coding (1,712–1,713/2,340 nt)	*BIF_01789* ←
1885792	+C	Coding (1,708/2,340 nt)	*BIF_01789* ←
1885854	Δ1 bp	Coding (1,646/2,340 nt)	*BIF_01789* ←
1893674	2 bp→TGC	Coding (2,445–2,446/2,673 nt)	*BIF_00866* →
1893713	+G	Coding (2,484/2,673 nt)	*BIF_00866* →
1893740	+G	Coding (2,511/2,673 nt)	*BIF_00866* →
1893947	A→C	Intergenic (+45/+15)	*BIF_00866* → / ← *BIF_00684*
1893952	3 bp→26 bp	Intergenic (+50/+8)	*BIF_00866* → / ← *BIF_00684*
1903558	34 bp→73 bp	Intergenic (+2/+61)	*BIF_00971* → / ← *BIF_01033*
1913433	C→T	Intergenic (+33/+59)	*BIF_02085* → / ← *BIF_01409*
1914632	+AAGGGGCGCCG	Coding (60/1,200 nt)	*BIF_01409* ←
1914636	A→G	V19A (GTA→GCA)	*BIF_01409* ←
1932289	+G	Intergenic (+61/+53)	*BIF_02266* → / ← *BIF_00333*
1932291	+T	Intergenic (+63/+51)	*BIF_02266* → / ← *BIF_00333*
1932292	+G	Intergenic (+64/+50)	*BIF_02266* → / ← *BIF_00333*
1932294	1 bp→GC	Intergenic (+66/+48)	*BIF_02266* → / ← *BIF_00333*
1932295	+CA	Intergenic (+67/+47)	*BIF_02266* → / ← *BIF_00333*
1932335	+CGCCT	Intergenic (+107/+7)	*BIF_02266* → / ← *BIF_00333*

a The nucleotide position column indicates the nucleotide position in GenBank accession number NC_017214.1, the mutation column indicates the genetic variant, the annotation column shows the effect of a variant on a gene (for intergenic variants, the numbers in parentheses indicate the variant’s position relative to the flanking genes, with positive numbers indicating that the variant is downstream and negative numbers indicating that the variant is upstream; for coding variants, the numbers in parentheses indicate the variant’s position in the affected gene and the gene's length), and the gene(s) column shows the locus tag(s) of the gene(s) in which or between which the variant occurs.

### Data availability.

The genome sequence has been deposited in NCBI GenBank with accession number CP001853.2. The raw reads have been deposited in the SRA under BioProject number PRJNA42883 with accession numbers SRX9857028 (MiSeq) and SRX9857029 (MinION).
